# S78. EXAMINING SEMANTIC AND EPISODIC MEMORY IN SCHIZOPHRENIA USING THE HOPKINS VERBAL LEARNING TASK

**DOI:** 10.1093/schbul/sby018.865

**Published:** 2018-04-01

**Authors:** Erica Neill, Susan Rossell

**Affiliations:** 1 University of Melbourne; 2 Swinburne University

## Abstract

**Background:**

Schizophrenia is associated with deficits in both episodic and semantic memory however, our understanding of how the deficits in each system independently contribute to overall memory performance is poorly understood.

The Hopkins Verbal Learning Task (HVLT) is a memory task using a single word list. To perform the task successfully, participants need to use both episodic and semantic abilities. Both episodic and semantic clustering scores can be calculated which provide nuanced information about the memory encoding and retrieval techniques used by those performing the task.

**Methods:**

Sixty schizophrenia patients and sixty healthy controls were compared in their performance on the HVLT. In addition to analysing immediate recall, learning slope, delayed recall and recognition, semantic and episodic clustering were also compared. Further, given the link between thought disorder and semantic function, this symptom was correlated with memory performance measures.

**Results:**

The schizophrenia group demonstrated worse performance across learning trials, delayed recall, and recognition indicating a generalised memory problem. Clustering scores were used to probe into semantic and episodic function specifically. The schizophrenia group demonstrated normal episodic clustering in the face of significantly impaired semantic clustering. Further, semantic clustering performance positively correlated with all general memory measures whilst episodic clustering did not. Finally, thought disorder did not correlate with any HVLT performance measure apart from semantic clustering.

**Discussion:**

It is difficult to tease apart the contributions of semantic and episodic memory impairments to poor overall memory function in schizophrenia. In this study, we have first demonstrated intact episodic clustering in the face of impaired semantic clustering. Then, by correlating semantic and episodic clustering scores with general memory performance measures, we were able to demonstrate that semantic memory performance is more significantly related to overall memory performance than episodic performance. Finally, this result supports the specificity of the relationship between thought disorder and semantic memory impairment.

